# Commuting and work-related accidents among employed Brazilians, National Survey of Health 2013 and 2019

**DOI:** 10.1590/1980-549720230006.supl.1

**Published:** 2023-04-21

**Authors:** Deborah Carvalho Malta, Regina Tomie Ivata Bernal, Nádia Machado de Vasconcelos, Adalgisa Peixoto Ribeiro, Lêda Lúcia Couto de Vasconcelos, Elaine Leandro Machado

**Affiliations:** I Universidade Federal de Minas Gerais, School of Nursing, Department of Maternal and Child Nursing and Public Health – Belo Horizonte (MG), Brazil.; II Universidade Federal de Minas Gerais, School of Nursing, Gradute Program in Nursing – Belo Horizonte (MG), Brazil.; III Universidade Federal de Minas Gerais, School of Medicine, Graduate Program in Public Health – Belo Horizonte (MG), Brazil.; IV Universidade Federal de Minas Gerais, School of Medicine, Department of Preventive and Social Medicine – Belo Horizonte (MG), Brazil.; V Universidade Federal de Sergipe, Department of Medicine – Lagarto (SE), Brazil.

**Keywords:** Occupational accidents, Occupational accidents registry, Health surveys, Epidemiological monitoring

## Abstract

**Objective:**

To analyze the prevalence of work-related accidents, according to sociodemographic and occupational variables, in 2013 and 2019.

**Methods:**

Cross-sectional study using data from the National Survey of Health (PNS) 2013 and 2019. Typical work accidents (WA), commuting accidents (CA), and Total Work Accidents (TWA) were evaluated. Prevalence values and 95% confidence intervals (95%CI) of TWA in 2013 and 2019 were estimated according to the explanatory variables and for Federative Units and capitals. In 2019, the prevalence and 95%CI according to explanatory variables were estimated using prevalence ratios (PR), both crude and adjusted for sex and age group.

**Results:**

TWA prevalence decreased from 4.96% (95%CI 4.55–5.38) in 2013 to 4.13% (95%CI 3.80–4.46) in 2019. In 2013, the state of Pará prevailed in TWA, and the state of Mato Grosso in 2019. The prevalence of WA and CA in 2019 were: 2.64% (95%CI 2.37–2.91) and 1.60% (95%CI 1.40–1.80). In 2019, the prevalence for TWA were higher for men (PR: 1.92; 95%CI 1.62–2.27); in the 18-29 age group (PR: 2.71; 95%CI 1.99–3.68); people with elementary school and some high school (PR: 2.09; 95%CI 1.57–2.78); and Black individuals (PR: 1.43; 95%CI 1.12–1.84). People without formal employment contract had a lower prevalence of TWA (PR: 0.77; 95%CI 0.66–0.90). WA was higher in rural areas (PR: 1.32; 95%CI 1.09–1.60).

**Conclusion:**

There was a reduction in TWA between 2013 and 2019. Men, young people, Black people, and individuals with lower level of education, residents in rural areas had higher prevalence of WA in 2019, demonstrating a relationship between health-disease-accident processes.

## INTRODUCTION

Work-related accident is characterized as occurring while working, regardless of the employment relationship (formal or informal), and leads to bodily injury or functional disturbance that results in death, loss, or temporary or permanent reduction of work capacity^1^. Total work accidents (TWA) can be divided into two categories: typical work accidents (WA), which occur in the workplace; and commuting accidents (CA), those suffered by the worker on their way from the residence to the workplace or vice versa, and may happen in any means of transportation, either public or private transport^1^.

According to estimates of the International Labor Organization (ILO), approximately 2.3 million deaths worldwide annually occur due to work-related accidents or illnesses^2^. Work-related illnesses are estimated to reach 160 million victims annually, with substances hazardous to health causing approximately 650 thousand deaths per year^2^. There are also approximately 340 million TWA per year^2^, and the industry and construction sectors have the highest accident rates, predominantly affecting workers at extreme ages^3^. Even with such high numbers, the ILO highlights the high underreporting of occupational accidents and diseases, including fatal accidents, due to poor working conditions in most countries^2^.

In Brazil, between 2012 and 2021, 6.2 million work-related accident communications (WAC) were recorded. Moreover, in those 10 years, 22,954 people died in work accidents. In 2021 alone, 571,800 accidents and 2,487 deaths associated with work were reported, an increase of 30% compared with 2020^4^.

Work-related accidents are preventable and have a great impact on productivity and the economy in addition to causing suffering to workers^5^. The costs of accidents include direct costs, with indemnities, expenses regarding medical care, judicial costs, social security costs; and indirect ones, with losses in production, reduced productivity, hours absent from work, among others. Between 2012 and 2021, the Brazilian social security expenditure exceeded BRL 120 billion with accident expenses alone, and approximately 469 million working days were lost^4^. This scenario reflects the low effectiveness of policies and programs to prevent health problems at work^5^.

Information on accidents and occupational or work-related diseases is fundamental for recognizing the urgency and prioritization of actions aimed at improving the working and health conditions of workers. The Brazilian Ministry of Labor and Social Security gathers information on work-related accidents according to WAC records and accident benefits granted by the National Social Security Institute (*Instituto Nacional do Seguro Social* – INSS)^6^. However, these data only cover employees with a formal employment contract, slightly more than half of the population employed in the country. The population with informal work has grown, especially after 2015, due to the economic crisis, the austerity policies implemented^7^, and the labor reform that took place in 2017, which relaxed labor laws, reducing the proportion of workers with formal work relationship in the country^8,9^.

The 2013 National Survey of Health (*Pesquisa Nacional de Saúde* – PNS) raised questions about work accidents in its questionnaire and showed that CA accounted for 30% of TWA^10^. In 2019, these questions were repeated, but the Brazilian Institute of Geography and Statistics (*Instituto Brasileiro de Geografia e Estatística* – IBGE) did not include commuting accidents in the estimation of the TWA prevalence disclosed, resulting in values lower than the actual ones^11^. Thus, the present study becomes paramount to re-estimate the prevalence of TWA in 2019, including both typical work accidents and commuting accidents and allowing a comparison with PNS 2013.

Therefore, the aim of this study was to analyze the prevalence of work-related accidents, according to sociodemographic and occupational variables, in 2013 and 2019.

## METHODS

### Study design and data source

This is a cross-sectional study that analyzed data from PNS 2013 and 2019^11–14^. The PNS sample was drawn by clustering sampling in three stages of selection: census tracts or set of sectors (primary units), households (secondary units), and adult residents (tertiary units)^11,12^.

In 2013, residents were interviewed in 64,348 households, and 60,202 interviews were conducted with adults (18 years or older). Of these, 36,442 individuals reported being employed during the reference week (from July 21 to July 27, 2013). In 2019, the expected sample of PNS 2019 was 108,525 households and data were collected in 94,114 households. Of these, 52,475 individuals, aged 18 years and over, reported being employed in the reference week (from July 21 to July 27, 2019)^11,12^.

### Variables

For the outcome variables, the present study considered work-related accident as the one that occurs while working and commuting from the residence to work, or vice versa, as recommended by Law No. 8.213 of July 24, 1991^1^. For the analyzed indicators, the considered denominator was the number of individuals who reported being employed in the reference week. The analyzed outcome variables were:

1. Work accidents (WA) — percentage of individuals aged 18 years or older who have been involved in work accidents in the last 12 months;
2. Commuting accidents (CA) — percentage of individuals aged 18 years or older who were involved in traffic accidents in the last 12 months when they were working, going to or returning from work;
3. Total work accidents (TWA) — percentage of individuals aged 18 years or older who were involved in total work accidents (accidents at work or while commuting to work).

The analyzed explanatory variables were:

- sociodemographic variables: sex (men and women), age group (18 to 29, 30 to 39, 40 to 59, 60 years or over), level of education (without formal education and some elementary school; elementary school and some high school; high school and some college; college degree), race/skin color (White, Black, mixed-race, other [Asian and Indigenous peoples]), place of residence (urban or rural), housing region (North, Northeast, Midwest, Southeast, and South);
- occupation variables: type of work (domestic worker, military, private sector, public sector, employer, independent business owner, unpaid), formal employment contract (yes or no).

### Statistical analysis

For 2013 and 2019, the prevalence values and confidence intervals of 95% (95%CI) of TWA were estimated, according to the explanatory variables. To evaluate the differences in prevalence in the two years, the crude (PRc) prevalence ratios (PR) were estimated using the Poisson regression model with robust variation. The prevalence and 95%CI of TWA were also estimated according to Federative Units (FU) and capitals in both years.

For PNS 2019, the prevalence and 95%CI of all indicators (WA, CA, and TWA) were estimated, according to the explanatory variables, and PRc, by the Poisson regression model with robust variation. PR adjusted by sex and age group (PRadj) were estimated because they are potential confounders^2,10^.

The data were analyzed in the Stata 16.0 software by the survey module, which considers the effects of complex sampling and sample weights, with the correction of nonresponse and adjustments of population totals.

### Ethical aspects

All participants gave their consent at the time of the interview. The research was approved by the National Commission of Ethics in Research for Human Beings of the Brazilian Ministry of Health (Opinions No. 328.159, for the 2013 edition, and No. 3.529.376, for the 2019 edition).

## RESULTS

When comparing the prevalence of TWA in 2013 and 2019, we observed a reduction from 4.96% (95%CI 4.55–5.38) to 4.13% (95%CI 3.80–4.46) (PRc: 0.83; 95%CI 0.71–0.95). This reduction was significant among men (PRc: 0.85; 95%CI 0.74–0.98), in the age groups 30 to 39 years (PRc: 0.75; 95%CI 0.62–0.92) and 60 years or over (PRc: 0.62; 95%CI 0.40–0.96), for White (PRc: 0.80; 95%CI 0.66–0.96) and Black individuals (PRc: 0.69; 95%CI 0.49–0.97) and residents in the urban area (PRc: 0.84; 95%CI 0.73–0.95) (Table 1).

**Table 1:** Prevalence, crude prevalence ratio, and 95% confidence interval of employed adults who were involved in work-related accidents in the last 12 months, according to sociodemographic variables. National Survey of Health 2013 and 2019, Brazil.

Variables		Total work accidents		
		2013 (A) (n=36,442) % (95%CI)	2019 (B) (n=52,475) % (95%CI)	PRc (B/A) % (95%CI)
**Total**		4.96 (4.55–5.38)	4.13 (3.80–4.46)	**0.83 (0.71**–**0.95)**
Sex				
	Men	6.16 (5.51–6.82)	5.24 (4.76–5.73)	**0.85 (0.74–0.98)**
	Women	3.36 (2.89–3.84)	2.73 (2.34–3.13)	0.81 (0.66–1.00)
Age group (years)				
	18 to 29	5.91 (5.04–6.79)	5.78 (4.92–6.65)	0.98 (0.79–1.21)
	30 to 39	5.41 (4.64–6.19)	4.07 (3.50–4.65)	**0.75 (0.62–0.92)**
	40 to 59	4.19 (3.60–4.78)	3.65 (3.18–4.12)	0.87 (0.72–1.06)
	60 or over	3.49 (2.31–4.67)	2.17 (1.59–2.75)	**0.62 (0.40**–**0.96)**
Level of education				
	No formal education/some elementary school	5.52 (4.81–6.22)	4.64 (4.07–5.22)	0.84 (0.71–1.00)
	Elementary school/some high school	6.41 (5.13–7.69)	5.63 (4.52–6.77)	0.88 (0.66–1.17)
	High school/some college	4.91 (4.15–5.66)	4.18 (3.63–4.74)	0.85 (0.70–1.05)
	College degree	2.66 (1.92–3.39)	2.28 (1.82–2.75)	0.86 (0.61–1.21)
Race/skin color				
	White	4.24 (3.67–4.80)	3.38 (2.95–3.82)	**0.80 (0.66**–**0.96)**
	Black	7.23 (5.39–9.07)	5.00 (3.88–6.11)	**0.69 (0.49–0.97)**
	Mixed-race	5.34 (4.73–5.96)	4.71 (4.20–5.23)	0.88 (0.75–1.04)
	Others	4.03 (1.89–6.16)	2.41 (0.76–4.07)	0.60 (0.25–1.43)
Place of residence				
	Urban	4.82 (4.37–5.28)	4.03 (3.67–4.39)	**0.84 (0.73**–**0.95)**
	Rural	5.96 (5.02–6.89)	4.85 (4.22–5.48)	0.81 (0.66–1.00)

Missing data were not presented. Values in bold are statistically significant (p<0.05); A: 2013; B: 2019; 95%CI: 95% confidence interval; PRc: crude prevalence ratio.

**Figure 1:**
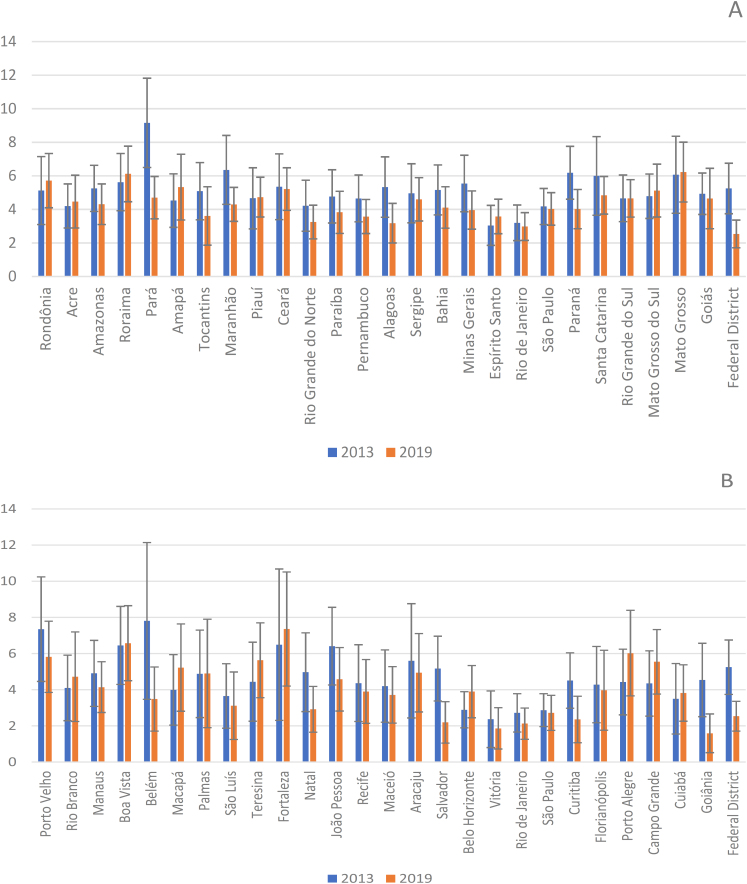
Prevalence of 2013 and 2019 of Brazilian employed adults who were involved in work-related accidents in the 12 months prior to the interview, in the states (A) and in the capitals (B). National Survey of Health 2013 and 2019, Brazil.

Among the federative units with the highest prevalence of TWA in 2013 were Pará (9.16%; 95%CI 6.5–11.82), Maranhão (6.35%; 95%CI 4.30–8.41), Paraná (6.18%; 95%CI 4.61–7.76), and Mato Grosso (6.07%; 95%CI 3.77–8.36). In 2019, the highest prevalence values were observed in Mato Grosso (6.23%; 95%CI 4.44–8.01), Roraima (6.12%; 95%CI 4.46–7.77), Rondônia (5.72%; 95%CI 4.10–7.33), and Amapá (5.33%; 95%CI 3.37–7.29) (Figure 1A). The analysis of PRc showed that there was a significant reduction in TWA between 2013 and 2019 for the states of Pará (PRc: 0.51; 95%CI 0.35–0.76), Alagoas (PRc: 0.60; 95%CI 0.36–0.98), Paraná (PRc: 0.65; 95%CI 0.44–0.96), and the Federal District (PRc: 0.48; 95%CI 0.31–0.75), in addition to the North (PRc: 0.67; 95%CI 0.52–0.85) and Northeast regions (PRc: 0.81; 95%CI 0.68–0.96) (Table 1, Supplementary Material).

Among Brazilian capitals, the highest prevalence values of TWA in 2013 were identified in Belém (7.81%; 95%CI 3.47–12.14), Porto Velho (7.35%; 95%CI 4.46–10.24), Fortaleza (6.49%; 95%CI 2.3–10.68), and Boa Vista (6.45%; 95%CI 4.29–8.61). In PNS 2019, higher prevalence values were found in Fortaleza (7.36%; 95%CI 4.2–10.51), Boa Vista (6.57%; 95%CI 4.5–8.65), Porto Alegre (6.02%; 95%CI 3.66–8.39), and Porto Velho (5.82%; 95%CI 3.85–7.79) (Figure 1B). We identified significant reductions between the two surveys in Belém (PRc: 0.45; 95%CI 0.21–0.95), Salvador (PRc: 0.42; 95%CI 0.23–0.80), and Goiânia (PRc: 0.35; 95%CI 0.15–0.79) (Table 2, Supplementary Material).

In 2019, 4.13% (95%CI 3.80–4.46) of the Brazilian employed population reported having suffered a work-related accident in the 12 months prior to the survey; 2.64% (95%CI 2.37–2.91) reported an accident at work and 1.60% (95%CI 1.40–1.80), while commuting. Workplace accidents were more frequent for men (PRadj: 1.76; 95%CI 1.42–2.20), those aged 18 to 29 years (PRadj: 2.22; 95%CI 1.53–3.23), 30 to 39 years (PRadj: 1.70; 95%CI 1.19–2.42), and 40 to 59 years (PRadj: 1.76; 95%CI 1.25–2.48), compared with those aged 60 years or over; those without formal education and with some elementary school (PRadj: 2.64; 95%CI 1.89–3.67), with elementary school and some high school (PRadj: 2.53; 95%CI 1.69–3.77), and with high school and some college (PRadj: 1.87; 95%CI 1.35–2.59), compared with those with college degree; and Black (PRadj: 1.53; 95%CI 1.12–2.08) and mixed-race people (PRadj: 1.34; 95%CI 1.07–1.69), compared with White people. WA in workers who live in rural areas were higher (PRadj: 1.32; 95%CI 1.09–1.60). Workers of other races (PRadj: 0.44; 95%CI 0.24–0.80) and those without a formal employment contract (PRadj: 0.78; 95%CI 0.63–0.96) had a lower frequency of this type of accident. Conversely, commuting accidents were more frequent among men (PRadj: 2.17; 95%CI 1.66–2.84), in the age groups 18 to 29 years (PRadj: 4.18; 95%CI 2.37–7.37) and 30 to 39 years (PRadj: 2.51; 95%CI 1.42–4.43), compared with the age group of 60 years or over; and in workers with elementary school and some high school (PRadj: 1.66; 95%CI 1.12–2.47), compared with those with college degree. Workers from the Southeast had a lower prevalence (PRadj: 0.59; 95%CI 0.41–0.83) compared with workers from the North. Domestic workers (PRadj: 0.24; 95%CI 0.09–0.62), compared with unpaid and those without a formal employment contract (PRadj: 0.75; 95%CI 0.58–0.97), had a lower frequency of this type of accident (Table 2).

**Table 2:** Prevalence, adjusted prevalence ratio, and 95%CI of employed adults who were involved in work-related accidents in the last 12 months, according to sociodemographic and occupational variables (n=52,475). National Survey of Health 2019, Brazil.

Variables			Work accidents		Commuting accidents		Total work accidents	
			% (95%CI)	PR_adj_ (95%CI)	% (95%CI)	PR_adj_ (95%CI)	% (95%CI)	PR_adj_(95%CI)
**Total**			2.64 (2.37–2.91)	–	1.60 (1.40–1.80)	–	4.13 (3.80–4.46)	–
Sociodemographic variables								
	Sex								
		Men	3.27 (2.86–3.67)	**1.76 (1.42–2.20)**	2.11 (1.81–2.41)	**2.17 (1.66–2.84)**	5.24 (4.76–5.73)	**1.92 (1.62–2.27)**
		Women	1.86 (1.52–2.19)	1.00 (-)	0.97 (0.74–1.19)	1.00 (-)	2.73 (2.34–3.12)	1.00 (-)
	Age group (years)							
		18 to 29	3.31 (2.61–4.01)	**2.22 (1.53–3.23)**	2.78 (2.20–3.35)	**4.18 (2.37–7.37)**	5.78 (4.92–6.65)	**2.71 (1.99–3.68)**
		30 to 39	2.50 (2.04–2.97)	**1.70 (1.19–2.42)**	1.65 (1.29–1.99)	**2.51 (1.42–4.43)**	4.07 (3.50–4.65)	**1.93 (1.43–2.60)**
		40 to 59	2.59 (2.18–3.00)	**1.76 (1.25–2.48)**	1.11 (0.86–1.36)	1.70 (0.96–3.01)	3.65 (3.18–4.12)	**1.73 (1.29–2.32)**
		60 or over	1.51 (1.05–1.97)	1.00 (-)	0.68 (0.32–1.03)	1.00 (-)	2.17 (1.59–2.75)	1.00 (-)
	Level of education							
		No formal education/some elementary school	3.32 (2.83–3.84)	**2.64 (1.89–3.67)**	1.44 (1.13–1.74)	1.40 (0.96–2.04)	4.64 (4.07–5.22)	**2.02 (1.58–2.58)**
		Elementary school/some high school	3.55 (2.55–4.58)	**2.53 (1.69–3.77)**	2.24 (1.66–2.81)	**1.66 (1.12–2.47)**	5.63 (4.52–6.77)	**2.09 (1.57–2.78)**
		High school/some college	2.56 (2.15–2.98)	**1.87 (1.35–2.59)**	1.76 (1.39–2.14)	**1.35 (0.93–1.97)**	4.18 (3.63–4.74)	**1.60 (1.25–2.04)**
		College degree	1.24 (0.89–1.60)	1.00 (-)	1.05 (0.74–1.36)	1.00 (-)	2.28 (1.82–2.75)	1.00 (-)
	Race/skin color							
		White	2.15 (1.79–2.51)	1.00 (-)	1.36 (1.08–1.63)	1.00 (-)	3.38 (2.95–3.82)	1.00 (-)
		Black	3.35 (2.48–4.25)	**1.53 (1.12–2.08)**	1.81 (1.20–2.41)	1.27 (0.87–1.86)	5.00 (3.88–6.11)	**1.43 (1.12–1.84)**
		Mixed-race	2.99 (2.56–3.44)	**1.34 (1.07–1.69)**	1.80 (1.49–2.11)	1.23 (0.94–1.60)	4.71 (4.20–5.23)	**1.32 (1.12–1.57)**
		Others	0.96 (0.40–1.51)	**0.44 (0.24–0.80)**	1.54 (0.03–3.11)	1.08 (0.38–3.08)	2.41 (0.76–4.07)	0.69 (0.34–1.41)
	Place of residence							
		Urban	2.51 (2.21–2.80)	1.00 (-)	1.64 (1.42–1.86)	1.00 (-)	4.03 (3.67–4.39)	1.00 (-)
		Rural	3.56 (3.03–4.10)	**1.32 (1.09–1.60)**	1.35 (0.98–1.71)	0.74 (0.54–1.01)	4.85 (4.22–5.48)	1.11 (0.94–1.30)
	Regions							
		North	2.46 (2.01–2.91)	1.00 (-)	2.43 (1.89–2.97)	1.00 (-)	4.69 (4.00–5.38)	1.00 (-)
		Northeast	2.57 (2.18–2.95)	1.07 (0.84–1.35)	1.75 (1.42–2.07)	0.75 (0.56–1.00)	4.16 (3.69–4.64)	0.91 (0.76–1.10)
		Southeast	2.60 (2.09–3.10)	1.11 (0.85–1.45)	1.31 (0.95–1.66)	**0.59 (0.41–0.83)**	3.80 (3.19–4.42)	0.87 (0.70–1.07)
		South	2.85 (2.30–3.38)	1.21 (0.93–1.57)	1.68 (1.27–2.10)	0.75 (0.54–1.05)	4.47 (3.80–5.14)	1.01 (0.82–1.24)
		Midwest	2.90 (2.22–3.58)	1.21 (0.90–1.63)	1.90 (1.41–2.39)	0.83 (0.59–1.16)	4.66 (3.77–5.55)	1.03 (0.81–1.31)
Occupational variables								
	Type of work							
		Domestic worker	1.80 (1.25–2.35)	1.72 (0.75–3.96)	0.39 (0.20–0.58)	**0.24 (0.09–0.62)**	2.18 (1.60–2.76)	0.79 (0.41–1.52)
		Military	1.98 (0.22–4.19)	1.11 (0.28–4.36)	3.41 (0.92–5.89)	0.95 (0.32–2.82)	5.30 (2.02–8.58)	1.02 (0.43–2.42)
		Private sector	3.28 (2.78–3.78)	2.15 (0.97–4.79)	2.06 (1.71–2.42)	0.70 (0.31–1.58)	5.18 (4.60–5.78)	1.19 (0.64–2.19)
		Public Sector	1.96 (1.30–2.62)	1.52 (0.65–3.58)	0.97 (0.69–1.25)	0.47 (0.20–1.10)	2.93 (2.22–3.65)	0.85 (0.44–1.62)
		Employer	1.75 (0.95–2.55)	1.21 (0.49–3.02)	1.01 (0.01–2.01)	0.43 (0.12–1.49)	2.71 (1.46–3.97)	0.69 (0.32–1.48)
		Independent business owner	2.31 (1.97–2.66)	1.61 (0.72–3.58)	1.43 (1.11–1.74)	0.58 (0.25–1.34)	3.61 (3.15–4.08)	0.91 (0.49–1.69)
		Unpaid	1.27 (0.28–2.26)	1.00 (-)	2.42 (0.46–4.38)	1.00 (-)	3.59 (1.42–5.76)	1.00 (-)
	Formal employment contract							
		Yes	3.19 (2.64–3.74)	1.00 (-)	2.06 (1.65–2.47)	1.00 (-)	5.09 (4.44–5.75)	1.00 (-)
		No*	2.31 (2.04–2.58)	**0.78 (0.63–0.96)**	1.33 (1.12–1.53)	**0.75 (0.58–0.97)**	3.56 (3.22–3.89)	**0.77 (0.66–0.90)**

Missing data were not presented.

* Missing data were classified as category “No”; values in bold are statistically significant (p<0.05). PR_adj:_ prevalence ratio adjusted by sex and age; 95%CI: 95% confidence interval.

With regard to TWA, they were positively associated with: men (PRadj: 1.92; 95%CI 1.62–2.27), younger age groups ([18 to 29 years – PRadj: 2.71; 95%CI 1.99–3.68], [30 to 39 years – PRadj: 1.93; 95%CI 1.43–2.60], [40 to 59 years – PRadj: 1.73; 95%CI 1.29–2.32]), in relation to 60 years or over; lower levels of education ([without formal education and some elementary school – PRadj: 2.02; 95%CI 1.58–2.58], [elementary school and some high school – PRadj: 2.09; 95%CI 1.57–2.78], [high school and some college – PRadj: 1.60; 95%CI 1.25–2.04]), in relation to college degree; and Black (PRadj: 1.43; 95%CI 1.12–1.84) and mixed-race skin colors (PRadj: 1.32; 95%CI 1.12–1.57), compared with White individuals. Conversely, TWA was negatively associated with the absence of a formal employment contract (PRadj: 0.77; 95%CI 0.66–0.90) in relation to those who had it (Table 2).

## DISCUSSION

In this study, we verified a reduction in TWA between 2013 and 2019. In 2019, the main victims of TWA were men, young people, Black and mixed-race individuals, those with lower level of education and who had a formal employment contract. Typical work accidents were more frequent among rural workers, and commuting accidents had no difference between urban and rural. In 2019, the report of work-related accidents occurred in about 4% of the active population, and commuting accidents corresponded to about 38% of the total accidents. In 2013, the state of Pará prevailed in TWA and, in 2019, the highest prevalence was in Mato Grosso.

We verified disparities between FU, with worse results in Pará, Roraima, Mato Grosso, and Mato Grosso do Sul. Data from the Brazilian Ministry of Social Security and Social Assistance highlight the worst results of work accidents in the North and Northeast states of the country^13^. However, in the comparison between 2013 and 2019, these two regions showed significant reductions in prevalence, while states of the Midwest prevailed. This high prevalence of TWA in the Midwest can be explained by the large number of rural workers and the high occurrence of work accidents in the rural area. Occupational accidents involving machinery and equipment result in amputations and other serious injuries in a frequency 15 times higher than other causes, generating three times more fatal accidents than the general average^4^.

Mato Grosso is the second state with the highest prevalence of accident notification, with 150 cases per 10 thousand workers, behind Rio Grande do Sul. The state recorded over 10 thousand work-related accidents in 2021 and, in the last 10 years (2012 to 2021), most of the accidents were caused by the operation of machinery and equipment (15%)^4^. Regarding mortality, Mato Grosso occupies the first position with 14 deaths per 10 thousand workers^4^. This accident occurrence profile may be related to the productive profile of the state, in which agriculture employs about 70% of the economically active population of the municipalities^14^.

This scenario shows the need for adequate training of workers and greater supervision regarding compliance with safety standards, especially Regulatory Standard No. 12 (NR-12) of the Brazilian Ministry of Labor and Social Security^15^, on Safety at Work in Machinery.

The PNS 2019 identified that about 38% of TWA occurred while commuting to the workplace, a percentage higher than that found in PNS 2013 (30%)^10^. According to the provisions of Art. 21, Item IV, Subitem *d*, of Law No 8.213/1991, the commuting accident guarantees the injured worker the same rights as a typical work accident^1^. However, on November 12, 2019, the Provisional Presidential Decree (*Medida Provisória* – MP) No. 905^16^ established that commuting accidents should not confer on the employee the same rights as work accidents. Because it is provisional, this MP was not converted into law and expired on April 21, 2020. Thereafter, the intended flexibility also ceased to exist and, consequently, commuting and the work-related accidents are equal before law^1^.

Data from emergency care for work-related injuries also indicate that, among work accidents, 31.3% were related to transportation^17^. It is worth highlighting that the increase in activities involving motorcycles, used for transportation/commuting between work and residence and as a work instrument (motorcycle taxi, freight shipping, among others), may have contributed to the increase in these accidents. In addition, there was a 53% increase in motorcycle deaths from 1990 to 2019, and the mortality rate for men increased from 7.3/100 thousand (1990) to 11.7/100 thousand inhabitants (2019)^18^.

Findings of PNS 2019 are in agreement with other studies that indicate that men, young people (18 to 39 years old), mixed-race and Black individuals, workers with lower level of education and lower income are more exposed to accidents^3,17,19–21^.

According to the literature, the sectors with the highest WA rates are industry and construction, which employ more male workers, with low qualifications and lower levels of education — therefore with greater social vulnerability and accident occurrence^4,22–24^. The greater accident occurrence among young people may be related to less experience in the profession, lower qualification, and exposure to high-risk jobs such as construction^25^.

The study pointed out that the prevalence of WA in rural areas was about 30% higher than in urban areas; nonetheless, there were no differences concerning CA between urban and rural areas. Previous studies have indicated a higher prevalence of work accidents in the rural area^25,26^, which can be explained by the specificities of the present study, with risks inherent in the management of animals, machinery, exposure to sharp-edged, contaminated materials and, often, these risks are increased by the workers’ low level of education and training. The importance of PNS for this record is noteworthy, because notifications of accidents in the urban area are historically higher, which is due to greater access to healthcare services in this area, combined with informal work in the field, leading to underreporting and minimization of the risks of accidents^26^.

The highest occurrence of TWA in workers with formal employment contract is in accordance with previous studies^27,28^. However, other studies report a higher prevalence of TWA among workers with an informal employment contract (54.3%)^19^. Therefore, these results should be better investigated, considering other variables of the health-disease-work process.

A study comparing official data from the Brazilian Ministry of Social Security and data released by the PNS in 2013 verified that PNS found seven times more accidents at work than the data on accidents recorded by Social Security^13^. Ministry data include only workers with formal employment relationship, excluding informal workers^13,17^, in such a way that monitoring TWA by the PNS becomes paramount.

Nevertheless, since 2015, Brazil has experienced a crisis scenario for workers, with increased unemployment, reduced protection guaranteed by public policies, and implementation of austerity measures^7^. The Labor Reform, sanctioned on July 13, 2017 by President Michel Temer, Law No. 13.467^29^, relaxed labor relations, increased the number of workers in informality, and precarious work^8^. The large share of informal workers is not considered in official statistics, which can make TWA even more underreported^30^.

Finally, we emphasize that data from the PNS 2019 were collected in the year before the COVID-19 pandemic. The health crisis may have affected the occurrence of TWA, as the increase in informality, in addition to delivery workers who use bicycles and motorcycles, may have directly impacted work safety^31,32^.

Among the limitations of the study, we point out those inherent in cross-sectional studies such as limitation in the determination of causality. Furthermore, the data are self-reported, and there can be differences in the interviewees’ understanding, recall bias, and under- or overestimation of the mentioned values, which may have influenced the results.

This is the first study comparing TWA data between PNS 2013 and 2019, enabling the recognition of the situation of this hazard in the country and its evolution in recent years. The sample size of representative coverage of the Brazilian adult population and the adopted research procedures strengthen the reliability of the data.

All in all, this study showed that there was a reduction in TWA in Brazil between 2013 and 2019, with important vulnerabilities to this hazard among men, mixed-race and Black individuals, young people and those with lower levels of education. Typical work accidents were up to 32% higher in rural areas and commuting accidents increased in 2019. Considering that labor rights and safety at work are part of the Sustainable Development Goals of the 2030 Agenda for Sustainable Development^33^, this study allows identifying priority groups for implementing agendas of actions aimed at preventing work-related accidents. Thus, there must be an intersectoral collaboration aimed not only at reducing costs for the economy and social security, but mainly at the well-being of workers and social equity.
