# Supplementary material for: Commuting and work-related accidents among employed Brazilians, National Survey of Health 2013 and 2019
Source: Rev Bras Epidemiol. 2023 Apr 21;26(Suppl 1):e230006. doi: 10.1590/1980-549720230006.supl.1 (PMC10176726; doi:10.1590/1980-549720230006.supl.1)
Supplement: Supplementary file 1 [file 1980-5497-rbepid-26-suppl1-e230006-suppl1.pdf]

Material suplementar

Tabela 1. Prevalência, razão de prevalência bruta e intervalo de confiança 95% de adultos ocupados que se envolveram em acidente de trabalho nos 12 meses anteriores à entrevista, segundo Estado de residência. Pesquisa Nacional de Saúde 2013 e 2019, Brasil

UF	Total Acidentes de Trabalho					
	**2013 (A) (n=36.442)**		**2019 (B) (n=52.475)**		**RP(B/A)**	**IC95%**
	**%**	**IC95%**	**%**	**IC95%**		
**Norte**	**7,04**	**(5,68-8,41)**	**4,69**	**(4,00-5,38)**	**0,67**	**(0,52-0,85)**
Rondônia	5,13	(3,10-7,15)	5,72	(4,10-7,33)	1,11	(0,69-1,80)
Acre	4,20	(2,89-5,52)	4,47	(2,89-6,04)	1,06	(0,66-1,70)
Amazonas	5,25	(3,88-6,63)	4,31	(3,10-5,52)	0,82	(0,56-1,20)
Roraima	5,62	(3,92-7,33)	6,12	(4,46-7,77)	1,09	(0,72-1,64)
Pará	9,16	(6,5-11,82)	4,70	(3,44-5,96)	0,51	(0,35-0,76)
Amapá	4,53	(2,93-6,12)	5,33	(3,37-7,29)	1,18	(0,70-1,97)
Tocantins	5,09	(3,38-6,79)	3,61	(1,87-5,35)	0,71	(0,39-1,28)
**Nordeste**	**5,13**	**(4,51-5,75)**	**4,16**	**(3,69-4,64)**	**0,81**	**(0,68-0,96)**
Maranhão	6,35	(4,30-8,41)	4,30	(3,29-5,31)	0,68	(0,45-1,01)
Piauí	4,67	(2,84-6,48)	4,73	(3,55-5,92)	1,01	(0,64-1,61)
Ceará	5,35	(3,39-7,31)	5,21	(3,94-6,48)	0,97	(0,63-1,51)
Rio Grande do Norte	4,22	(2,70-5,74)	3,25	(2,24-4,25)	0,77	(0,48-1,24)
Paraíba	4,77	(3,19-6,36)	3,83	(2,57-5,08)	0,80	(0,50-1,28)
Pernambuco	4,65	(3,26-6,05)	3,57	(2,56-4,59)	0,77	(0,51-1,16)
Alagoas	5,33	(3,53-7,13)	3,18	(2,00-4,36)	0,60	(0,36-0,98)
Sergipe	4,96	(3,20-6,72)	4,60	(3,31-5,89)	0,93	(0,59-1,47)
Bahia	5,16	(3,67-6,65)	4,11	(2,88-5,35)	0,80	(0,53-1,20)
**Sudeste**	**4,27**	**(3,52-5,02)**	**3,80**	**(3,19-4,42)**	**0,89**	**(0,70-1,13)**
Minas Gerais	5,54	(3,86-7,23)	3,96	(2,82-5,10)	0,71	(0,47-1,09)
Espírito Santo	3,04	(1,86-4,24)	3,58	(2,55-4,61)	1,18	(0,72-1,91)

Rio de Janeiro	3,20	(2,14-4,26)	2,98	(2,15-3,81)	0,93	(0,60-1,44)
São Paulo	4,18	(3,10-5,25)	4,03	(3,06-5,00)	0,96	(0,68-1,37)
**Sul**	**5,54**	**(4,56-6,52)**	**4,47**	**(3,80-5,14)**	**0,81**	**(0,64-1,02)**
Paraná	6,18	(4,61-7,76)	4,02	(2,85-5,19)	0,65	(0,44-0,96)
Santa Catarina	6,00	(3,65-8,34)	4,84	(3,72-5,96)	0,81	(0,51-1,28)
Rio Grande do Sul	4,66	(3,27-6,05)	4,66	(3,54-5,77)	1,00	(0,68-1,47)
**Centro-Oeste**	**5,20**	**(4,39-6,01)**	**4,66**	**(3,77-5,55)**	**0,90**	**(0,70-1,15)**
Mato Grosso do Sul	4,79	(3,46-6,11)	5,12	(3,55-6,70)	1,07	(0,71-1,62)
Mato Grosso	6,07	(3,77-8,36)	6,23	(4,44-8,01)	1,03	(0,64-1,65)
Goiás	4,93	(3,70-6,17)	4,65	(2,85-6,45)	0,94	(0,60-1,49)
Distrito Federal	5,25	(3,74-6,75)	2,54	(1,71-3,36)	0,48	(0,31-0,75)

Nota: % - percentual; RP - Razão de prevalência bruta; IC95% - Intervalo de confiança de 95%; A – 2013; B- 2019; valores em negrito – estatisticamente significativos (valor-p < 0,05).

Tabela 2. Prevalência, razão de prevalência bruta e intervalo de confiança 95% de pessoas de 18 anos ou mais de idade que se envolveram em acidente de trabalho nos 12 meses anteriores à entrevista, nas capitais. Pesquisa Nacional de Saúde 2013 e 2019, Brasil.

Capital	Total Acidentes de Trabalho					
	2013 (A) (n=36.442)		2019 (B) (n=52.475)		RP (B/A)	IC95%
	%	IC95%	%	IC95%		
Porto Velho	7,35	(4,46-10,24)	5,82	(3,85-7,79)	0,79	(0,47-1,34)
Rio Branco	4,10	(2,29-5,91)	4,72	(2,24-7,2)	1,15	(0,58-2,28)
Manaus	4,91	(3,08-6,73)	4,14	(2,74-5,55)	0,84	(0,51-1,40)
Boa Vista	6,45	(4,29-8,61)	6,57	(4,5-8,65)	1,02	(0,64-1,62)
Belém	7,81	(3,47-12,14)	3,49	(1,71-5,26)	0,45	(0,21-0,95)
Macapá	3,99	(2,04-5,94)	5,22	(2,81-7,64)	1,31	(0,66-2,58)
Palmas	4,88	(2,46-7,30)	4,90	(1,9-7,9)	1,00	(0,45-2,23)
São Luís	3,65	(1,87-5,44)	3,12	(1,25-4,98)	0,85	(0,39-1,85)
Teresina	4,44	(2,26-6,63)	5,63	(3,56-7,7)	1,27	(0,68-2,35)
Fortaleza	6,49	(2,3-10,68)	7,36	(4,2-10,51)	1,13	(0,52-2,47)
Natal	4,97	(2,79-7,15)	2,92	(1,65-4,18)	0,59	(0,31-1,09)
João Pessoa	6,41	(4,26-8,56)	4,58	(2,82-6,33)	0,71	(0,43-1,19)
Recife	4,36	(2,24-6,49)	3,90	(2,14-5,67)	0,89	(0,46-1,74)
Maceió	4,20	(2,20-6,20)	3,71	(2,15-5,28)	0,88	(0,47-1,68)
Aracaju	5,60	(2,44-8,76)	4,94	(2,77-7,11)	0,88	(0,43-1,82)
Salvador	5,17	(3,38-6,96)	2,20	(1,05-3,34)	0,42	(0,23-0,80)

Belo Horizonte	2,89	(1,89-3,89)	3,90	(2,45-5,34)	1,35	(0,81-2,25)
Vitória	2,37	(0,80-3,93)	1,86	(0,72-3,01)	0,79	(0,32-1,96)
Rio de Janeiro	2,72	(1,66-3,78)	2,13	(1,26-2,99)	0,78	(0,44-1,38)
São Paulo	2,87	(1,96-3,78)	2,72	(1,75-3,69)	0,95	(0,59-1,53)
Curitiba	4,51	(2,98-6,04)	2,36	(1,07-3,64)	0,52	(0,27-1,00)
Florianópolis	4,28	(2,17-6,39)	3,97	(1,76-6,18)	0,93	(0,44-1,96)
Porto Alegre	4,43	(2,61-6,24)	6,02	(3,66-8,39)	1,36	(0,77-2,39)
Campo Grande	4,35	(2,54-6,15)	5,55	(3,76-7,33)	1,28	(0,75-2,16)
Cuiabá	3,50	(1,55-5,45)	3,82	(2,26-5,38)	1,09	(0,54-2,19)
Goiânia	4,54	(2,51-6,57)	1,59	(0,52-2,66)	0,35	(0,15-0,79)
Distrito Federal	5,25	(3,74-6,75)	2,54	(1,71-3,36)	0,48	(0,31-0,75)

Nota: % - percentual; RP - Razão de prevalência bruta; IC95% - Intervalo de confiança de 95%; A – 2013; B- 2019; valores em negrito – estatisticamente significativos (valor-p < 0,05).

Tabela 3. Prevalência e razão de prevalência bruta, com respectivos intervalos de confiança 95%, de adultos ocupados que se envolveram em acidentes de trabalho nos 12 meses anteriores à entrevista, segundo variáveis sociodemográficas e de ocupação (n = 52.475). Pesquisa Nacional de Saúde 2019, Brasil.

Variáveis	Acidente no trabalho		Acidente no deslocamento		Acidente de trabalho total	
	% (IC95%)	RP (IC95%)	% (IC95%)	RP (IC95%)	% (IC95%)	RP (IC95%)
**Total**	2,64 (2,37-2,91)	-	1,60 (1,40-1,80)	-	4,13 (3,80-4,46)	-
**Variáveis sociodemográficas**						
**Sexo**						
Masculino	3,27 (2,86-3,67)	**1,76 (1,41-2,19)**	2,11 (1,81-2,41)	**2,18 (1,67-2,86)**	5,24 (4,76-5,73)	**1,92 (1,62-2,27)**
Feminino	1,86 (1,52-2,19)	1,00 (-)	0,97 (0,74-1,19)	1,00 (-)	2,73 (2,34-3,12)	1,00 (-)
**Faixa etária (anos)**						
18 a 29 anos	3,31 (2,61-4,01)	**2,19 (1,51-3,19)**	2,78 (2,20-3,35)	**4,09 (2,31-7,25)**	5,78 (4,92-6,65)	**2,66 (1,96-3,62)**
30 a 39 anos	2,50 (2,04-2,97)	**1,66 (1,16-2,36)**	1,65 (1,29-1,99)	**2,43 (1,37-4,29)**	4,07 (3,50-4,65)	**1,88 (1,39-2,53)**
40 a 59 anos	2,59 (2,18-3,00)	**1,72 (1,22-2,42)**	1,11 (0,86-1,36)	1,63 (0,93-2,91)	3,65 (3,18-4,12)	**1,68 (1,25-2,26)**
60 anos ou mais	1,51 (1,05-1,97)	1,00 (-)	0,68 (0,32-1,03)	1,00 (-)	2,17 (1,59-2,75)	1,00 (-)
**Escolaridade**						
Sem instrução e fundamental incompleto	3,32 (2,83-3,84)	**2,68 (1,94-3,70)**	1,44 (1,13-1,74)	1,37 (0,95-1,96)	4,64 (4,07-5,22)	**2,03 (1,60-2,58)**
Fundamental completo e médio incompleto	3,55 (2,55-4,58)	**2,86 (1,92-4,28)**	2,24 (1,66-2,81)	**2,13 (1,45-3,14)**	5,63 (4,52-6,77)	**2,47 (1,86-3,28)**

Médio completo e superior incompleto	2,56 (2,15-2,98)	**2,06 (1,49-2,86)**	1,76 (1,39-2,14)	**1,68 (1,17-2,43)**	4,18 (3,63-4,74)	**1,83 (1,44-2,34)**
Superior completo	1,24 (0,89-1,60)	1,00 (-)	1,05 (0,74-1,36)	1,00 (-)	2,28 (1,82-2,75)	1,00 (-)
**Raça/cor da pele**						
Branca	2,15 (1,79-2,51)	1,00 (-)	1,36 (1,08-1,63)	1,00 (-)	3,38 (2,95-3,82)	1,00 (-)
Preta	3,35 (2,48-4,25)	**1,56 (1,15-2,13)**	1,81 (1,20-2,41)	1,33 (0,91-1,95)	5,00 (3,88-6,11)	**1,48 (1,15-1,89)**
Parda	2,99 (2,56-3,44)	**1,39 (1,11-1,75)**	1,80 (1,49-2,11)	**1,33 (1,01-1,73)**	4,71 (4,20-5,23)	**1,39 (1,17-1,65)**
Outras	0,96 (0,40-1,51)	**0,45 (0,24-0,81)**	1,54 (0,03-3,11)	1,13 (0,40-3,21)	2,41 (0,76-4,07)	0,71 (0,35-1,44)
**Local de residência**						
Urbana	2,51 (2,21-2,80)	1,00 (-)	1,64 (1,42-1,86)	1,00 (-)	4,03 (3,67-4,39)	1,00 (-)
Rural	3,56 (3,03-4,10)	**1,42 (1,17-1,72)**	1,35 (0,98-1,71)	0,82 (0,61-1,11)	4,85 (4,22-5,48)	**1,20 (1,03-1,41)**
**Regiões**						
Norte	2,46 (2,01-2,91)	1,00 (-)	2,43 (1,89-2,97)	1,00 (-)	4,69 (4,00-5,38)	1,00 (-)
Nordeste	2,57 (2,18-2,95)	1,04 (0,83-1,32)	1,75 (1,42-2,07)	**0,72 (0,54-0,96)**	4,16 (3,69-4,64)	0,89 (0,74-1,07)
Sudeste	2,60 (2,09-3,10)	1,06 (0,81-1,38)	1,31 (0,95-1,66)	**0,54 (0,38-0,76)**	3,80 (3,19-4,42)	0,81 (0,65-1,01)
Sul	2,85 (2,30-3,38)	1,16 (0,89-1,50)	1,68 (1,27-2,10)	**0,69 (0,50-0,97)**	4,47 (3,80-5,14)	0,95 (0,77-1,18)
Centro-Oeste	2,90 (2,22-3,58)	1,18 (0,88-1,59)	1,90 (1,41-2,39)	0,78 (0,56-1,10)	4,66 (3,77-5,55)	0,99 (0,78-1,26)
**Variáveis Ocupacionais**						
**Tipo de trabalho**						
Trabalhador doméstico	1,80 (1,25-2,35)	1,42 (0,62-3,24)	0,39 (0,20-0,58)	**0,16 (0,06-0,41)**	2,18 (1,60-2,76)	0,61 (0,31-1,17)

Militar	1,98 (0,22-4,19)	1,56 (0,40-6,06)	3,41 (0,92-5,89)	1,41 (0,47-4,20)	5,30 (2,02-8,58)	1,48 (0,62-3,50)
Setor Privado	3,28 (2,78-3,78)	**2,58 (1,17-5,72)**	2,06 (1,71-2,42)	0,85 (0,37-1,95)	5,18 (4,60-5,78)	1,44 (0,78-2,67)
Setor Público	1,96 (1,30-2,62)	1,54 (0,66-3,61)	0,97 (0,69-1,25)	**0,40 (0,17-0,96)**	2,93 (2,22-3,65)	0,82 (0,42-1,57)
Empregador	1,75 (0,95-2,55)	1,38 (0,56-3,40)	1,01 (0,01-2,01)	0,42 (0,12-1,50)	2,71 (1,46-3,97)	0,75 (0,35-1,61)
Conta própria	2,31 (1,97-2,66)	1,82 (0,82-4,02)	1,43 (1,11-1,74)	0,59 (0,25-1,37)	3,61 (3,15-4,08)	1,01 (0,54-1,87)
Não remunerado	1,27 (0,28-2,26)	1,00 (-)	2,42 (0,46-4,38)	1,00 (-)	3,59 (1,42-5,76)	1,00 (-)
**Carteira de trabalho assinada**						
Sim	3,19 (2,64-3,74)	1,00 (-)	2,06 (1,65-2,47)	1,00 (-)	5,09 (4,44-5,75)	1,00 (-)
Não (*)	2,31 (2,04-2,58)	**0,72 (0,59-0,89)**	1,33 (1,12-1,53)	**0,65 (0,50-0,83)**	3,56 (3,22-3,89)	**0,70 (0,60-0,82)**

(*) Missing classificado como "Não"; valores em negrito – estatisticamente significativos (valor-p < 0,05)

Nota: % - percentual; RP – Razão de prevalência; IC95% - Intervalo de confiança de 95%
