# Supplementary material for: Commuting and work-related accidents among employed Brazilians, National Survey of Health 2013 and 2019
Source: Rev Bras Epidemiol. 2023 Apr 21;26(Suppl 1):e230006. doi: 10.1590/1980-549720230006.supl.1 (PMC10176726; doi:10.1590/1980-549720230006.supl.1)
Supplement: Translation

# Translation: Acidentes no deslocamento e no trabalho entre brasileiros ocupados, Pesquisa Nacional de Saúde 2013 e 2019

**DOI:** 10.1590/1980-549720230006.supl.1.1

**Authors:** Deborah Carvalho Malta, Regina Tomie Ivata Bernal, Nádia Machado de Vasconcelos, Adalgisa Peixoto Ribeiro, Lêda Lúcia Couto de Vasconcelos, Elaine Leandro Machado

**Affiliations:** I Universidade Federal de Minas Gerais, Escola de Enfermagem, Departamento de Enfermagem Materno Infantil e Saúde Pública – Belo Horizonte (MG), Brasil.; II Universidade Federal de Minas Gerais, Escola de Enfermagem, Programa de Pós-Graduação em Enfermagem – Belo Horizonte (MG), Brasil.; III Universidade Federal de Minas Gerais, Faculdade de Medicina, Programa de Pós-Graduação em Saúde Pública – Belo Horizonte (MG), Brasil.; IV Universidade Federal de Minas Gerais, Faculdade de Medicina, Departamento de Medicina Preventiva e Social – Belo Horizonte (MG), Brasil.; V Universidade Federal de Sergipe, Departamento de Medicina – Lagarto (SE), Brasil.

**Keywords:** Acidentes de trabalho, Notificação de acidentes de trabalho, Inquéritos epidemiológicos, Vigilância epidemiológica

## Abstract

**Objetivo:**

Analisar as prevalências de acidentes de trabalho, segundo variáveis sociodemográficas e ocupacionais, em 2013 e 2019.

**Métodos:**

Estudo transversal utilizando dados da Pesquisa Nacional de Saúde (PNS) 2013 e 2019. Avaliou-se os acidentes de trabalho típico (AT), de descolamento (AD) e de trabalho totais (ATT). As prevalências e os intervalos de confiança de 95% (IC95%) de ATT em 2013 e 2019 foram estimadas segundo as variáveis explicativas, unidades da Federação e capitais. Em 2019, foram calculadas as prevalências e IC95% segundo variáveis explicativas e razões de prevalência (RP) bruta e ajustada por sexo e faixa etária.

**Resultados:**

A prevalência de ATT passou de 4,96% (IC95% 4,55–5,38) em 2013 para 4,13% (IC95% 3,80–4,46) em 2019. Em 2013, o Pará liderou em prevalência de ATT e, em 2019, a maior prevalência foi em Mato Grosso. As prevalências de AT e AD em 2019 foram, respectivamente, 2,64% (IC95% 2,37–2,91) e 1,60% (IC95% 1,40–1,80). Em 2019, as prevalências para ATT foram mais elevadas para homens (RP: 1,92; IC95% 1,62–2,27); faixa etária de 18 a 29 anos (RP: 2,71; IC95% 1,99–3,68); pessoas com ensino fundamental completo/médio incompleto (RP: 2,09; IC95% 1,57–2,78); e pessoas de cor preta (RP: 1,43; IC95% 1,12–1, 84), e menor em pessoas sem carteira de trabalho (RP: 0,77; IC95% 0,66–0,90). AT foi maior na zona rural (RP: 1,32; IC95% 1,09–1,60).

**Conclusão:**

Houve redução dos ATT entre 2013 e 2019. Homens, jovens, pretos e indivíduos com menor escolaridade, trabalhadores da zona rural, apresentaram maiores prevalências de AT em 2019, demonstrando uma relação dos processos saúde-doença-acidente.

## INTRODUÇÃO

O acidente de trabalho é caracterizado como aquele que ocorre no exercício do trabalho, independentemente do vínculo (formal ou não), e produz lesão corporal ou perturbação funcional que resulte em morte, perda ou redução temporária ou permanente da capacidade para o trabalho^1^. Podem-se dividir os acidentes de trabalho totais (ATT) em duas categorias: os acidentes de trabalho típicos (AT), que ocorrem no local de trabalho; e os acidentes de deslocamento (AD), aqueles sofridos pelo trabalhador no percurso da residência para o local de trabalho ou vice-versa, podendo acontecer em qualquer meio de locomoção, seja no transporte público ou em veículo próprio^1^.

Segundo estimativas da Organização Internacional do Trabalho (OIT), anualmente ocorrem aproximadamente 2,3 milhões de mortes em todo o mundo devido a acidentes de trabalho ou doenças relacionadas ao trabalho^2^. Estima-se que as doenças relacionadas ao trabalho atinjam anualmente 160 milhões de vítimas, sendo que as substâncias perigosas à saúde causam aproximadamente 650 mil mortes por ano^2^. Há, ainda, aproximadamente 340 milhões de ATT por ano^2^, e os setores da indústria e da construção civil têm as taxas de acidentes mais elevadas, afetando predominantemente os trabalhadores nos extremos de idade^3^. Mesmo com números tão altos, a OIT destaca a elevada subnotificação de acidentes e doenças ocupacionais, incluindo acidentes fatais, devido às precárias condições de trabalho na maioria dos países^2^.

No Brasil, entre 2012 e 2021, houve o registro de 6,2 milhões de comunicações de acidente de trabalho (CAT). Ainda, nesses 10 anos, 22.954 pessoas morreram em acidentes de trabalho. Apenas em 2021, foram comunicados 571,8 mil acidentes e 2.487 óbitos associados ao trabalho, com aumento de 30% em relação a 2020^4^.

Os acidentes de trabalho são evitáveis e causam grande impacto sobre a produtividade e a economia, além de sofrimento ao trabalhador^5^. Os custos de acidentes incluem os diretos, com indenizações, gastos em assistência médica, custos judiciais, custos com previdência, e os indiretos, com perdas na produção, redução da produtividade, horas paralisadas, entre outras. O gasto previdenciário brasileiro, entre 2012 e 2021, ultrapassou os R$ 120 bilhões somente com despesas acidentárias, e foram perdidos cerca de 469 milhões de dias de trabalho^4^. Esse cenário reflete a baixa efetividade das políticas e dos programas de prevenção de agravos à saúde no trabalho^5^.

Informações sobre os acidentes e as doenças do trabalho ou relacionadas ao trabalho são fundamentais para o reconhecimento da urgência e a priorização das ações visando à melhoria das condições de trabalho e saúde dos trabalhadores. O Ministério do Trabalho e Previdência reúne informações sobre acidentes de trabalho, por meio dos registros de CAT e benefícios acidentários concedidos pelo Instituto Nacional do Seguro Social (INSS)^6^. Entretanto esses dados abrangem apenas os empregados com carteira assinada, pouco mais da metade da população empregada no país. A população em trabalho informal tem crescido, em especial após 2015, em função da crise econômica, das políticas de austeridade implantadas^7^ e da reforma trabalhista ocorrida em 2017, que flexibilizou as leis trabalhistas, reduzindo a proporção dos trabalhadores com vínculo formal no país^8,9^.

A Pesquisa Nacional de Saúde (PNS) de 2013 trouxe questões sobre acidentes de trabalho no seu questionário e mostrou que os AD responderam por 30% dos ATT^10^. Em 2019, essas questões foram repetidas, porém o Instituto Brasileiro de Geografia e Estatística (IBGE) não incluiu os acidentes de deslocamento no cálculo das prevalências de ATT divulgadas, resultando em valores menores que os reais^11^. Assim, o presente estudo torna-se fundamental para reestimar as prevalências de ATT em 2019, incluindo tanto os acidentes de trabalho típicos como os de deslocamento e permitindo a comparação com a PNS 2013.

Dessa forma, o objetivo do estudo atual foi analisar as prevalências de acidentes de trabalho, segundo variáveis sociodemográficas e ocupacionais, em 2013 e 2019.

## MÉTODOS

### Delineamento e fonte de dados

Este foi um estudo transversal que analisou dados da PNS de 2013 e 2019^11–14^. A amostra da PNS é por conglomerados em três estágios de seleção: setores censitários ou conjunto de setores (unidades primárias), domicílios (unidades secundárias) e moradores adultos (unidades terciárias)^11,12^.

Em 2013, foram entrevistados moradores em 64.348 domicílios, sendo realizadas 60.202 entrevistas com adultos (18 anos ou mais). Destes, 36.442 indivíduos referiram estar ocupados na semana de referência (de 21 a 27 de julho de 2013). Em 2019, a amostra prevista da PNS 2019 foi de 108.525 domicílios e os dados foram coletados em 94.114 domicílios. Destes, 52.475 indivíduos, com 18 anos e mais, referiram estar ocupados ou trabalhando na semana de referência (de 21 a 27 de julho de 2019)^11,12^.

### Variáveis

Para as variáveis de desfecho, o presente estudo considerou como acidente de trabalho aquele que ocorre no desempenho da função e no deslocamento da casa para o trabalho, ou vice-versa, como preconiza a Lei n° 8.213, de 24 de julho de 1991^1^. Para os indicadores analisados, o denominador considerado foi o número de indivíduos que referiram estar ocupados ou trabalhando na semana de referência. As variáveis de desfecho analisadas foram:

1. Acidentes de trabalho (AT) — porcentagem de indivíduos de 18 anos ou mais que se envolveram em acidentes de trabalho nos últimos 12 meses;
2. Acidentes ocorridos no deslocamento para o trabalho (AD) — porcentagem de indivíduos de 18 anos ou mais que se envolveram em acidentes de trânsito nos últimos 12 meses quando estavam trabalhando, indo ou voltando do trabalho;
3. Acidentes de trabalho total (ATT) — porcentagem de indivíduos de 18 anos ou mais que se envolveram em acidentes de trabalho total (acidente no local de trabalho ou no deslocamento para o trabalho).

As variáveis explicativas analisadas foram:

- variáveis sociodemográficas: sexo (masculino e feminino), faixa etária (de 18 a 29, de 30 a 39, de 40 a 59, 60 anos ou mais), escolaridade (sem instrução e fundamental incompleto; fundamental completo e médio incompleto; médio completo e superior incompleto; superior completo), raça/cor da pele (branca, preta, parda, outra [amarela e indígena]), local de residência (urbano ou rural), região de moradia (Norte, Nordeste, Centro-oeste, Sudeste e Sul);
- variáveis de ocupação: tipo de trabalho (trabalhador doméstico, militar, setor privado, setor público, empregador, conta própria, não remunerado), carteira de trabalho assinada (sim ou não).

### Análise estatística

Para 2013 e 2019, foram estimadas as prevalências e os intervalos de confiança de 95% (IC95%) dos ATT, segundo as variáveis explicativas. Para se avaliar as diferenças de prevalências nos dois anos, calcularam-se as razões de prevalência brutas (RP) por meio do modelo de regressão de Poisson com variação robusta. Foram calculadas, ainda, as prevalências e os IC95% dos ATT para as unidades da Federação (UF) e capitais em ambos os anos.

Para a PNS 2019, estimaram-se as prevalências e os IC95% de todos os indicadores (AT, AD e ATT), segundo as variáveis explicativas, e razões de prevalência (RP) brutas (RPb), por meio do modelo de regressão de Poisson com variação robusta. Optou-se por estimar a RP Ajustada (RPaj) por sexo e faixa etária, por serem potenciais confundidores^2,10^.

Os dados foram analisados no *software* Stata 16.0, por meio do módulo *survey*, que considera efeitos da amostragem complexa e os pesos amostrais, com a correção de não respostas e ajustes dos totais populacionais.

### Aspectos éticos

Todos os participantes informaram consentimento no momento da entrevista. As pesquisas foram aprovadas pela Comissão Nacional de Ética em Pesquisa para Seres Humanos do Ministério da Saúde (pareceres n° 328.159, para a edição de 2013, e n° 3.529.376, para a edição de 2019).

## RESULTADOS

Ao comparar a prevalência de ATT em 2013 e 2019, observou-se uma redução desse, passando de 4,96% (IC95% 4,55–5,38) para 4,13% (IC95% 3,80–4,46) (RPb: 0,83; IC95% 0,71–0,95). Essa redução foi significativa entre os homens (RPb: 0,85; IC95% 0,74–0,98), nas faixas etárias de 30 a 39 anos (RPb: 0,75; IC95% 0,62–0,92) e 60 anos e mais (RPb: 0,62; IC95% 0,40–0,96), para os indivíduos de cor da pele branca (RPb: 0,80; IC95% 0,66–0,96) e preta (RPb: 0,69; IC95% 0,49–0,97) e residentes na zona urbana (RPb: 0,84; IC95% 0,73–0,95) (Tabela 1).

**Tabela 1:** Prevalência, razão de prevalência bruta e IC95% de adultos ocupados que se envolveram em acidente de trabalho nos últimos 12 meses, segundo variáveis sociodemográficas. Pesquisa Nacional de Saúde 2013 e 2019, Brasil.

Variáveis		Acidentes de trabalho total		
		2013 (A) (n=36.442) % (IC95%)	2019 (B) (n=52.475) % (IC95%)	RPb (B/A) % (IC95%)
**Total**		4,96 (4,55–5,38)	4,13 (3,80–4,46)	**0,83 (0,71**–**0,95)**
Sexo				
	Masculino	6,16 (5,51–6,82)	5,24 (4,76–5,73)	**0,85 (0,74**–**0,98)**
	Feminino	3,36 (2,89–3,84)	2,73 (2,34–3,13)	0,81 (0,66–1,00)
Faixa etária (anos)				
	18 a 29	5,91 (5,04–6,79)	5,78 (4,92–6,65)	0,98 (0,79–1,21)
	30 a 39	5,41 (4,64–6,19)	4,07 (3,50–4,65)	**0,75 (0,62**–**0,92)**
	40 a 59	4,19 (3,60–4,78)	3,65 (3,18–4,12)	0,87 (0,72–1,06)
	60 ou mais	3,49 (2,31–4,67)	2,17 (1,59–2,75)	**0,62 (0,40**–**0,96)**
Escolaridade				
	Sem instrução/fundamental incompleto	5,52 (4,81–6,22)	4,64 (4,07–5,22)	0,84 (0,71–1,00)
	Fundamental completo/médio incompleto	6,41 (5,13–7,69)	5,63 (4,52–6,77)	0,88 (0,66–1,17)
	Médio completo e superior incompleto	4,91 (4,15–5,66)	4,18 (3,63–4,74)	0,85 (0,70–1,05)
	Superior completo	2,66 (1,92–3,39)	2,28 (1,82–2,75)	0,86 (0,61–1,21)
Raça/cor da pele				
	Branca	4,24 (3,67–4,80)	3,38 (2,95–3,82)	**0,80 (0,66**–**0,96)**
	Preta	7,23 (5,39–9,07)	5,00 (3,88–6,11)	**0,69 (0,49**–**0,97)**
	Parda	5,34 (4,73–5,96)	4,71 (4,20–5,23)	0,88 (0,75–1,04)
	Outras	4,03 (1,89–6,16)	2,41 (0,76–4,07)	0,60 (0,25–1,43)
Local de residência				
	Urbana	4,82 (4,37–5,28)	4,03 (3,67–4,39)	**0,84 (0,73**–**0,95)**
	Rural	5,96 (5,02–6,89)	4,85 (4,22–5,48)	0,81 (0,66–1,00)

Os dados faltantes não foram apresentados. Valores em negrito: estatisticamente significativos (p<0,05); A: 2013; B: 2019; IC95%: intervalo de confiança de 95%; RPb: razão de prevalência bruta.

Entre as unidades federativas com maior prevalência de ATT em 2013 estavam o Pará (9,16%; IC95% 6,5–11,82), o Maranhão (6,35%; IC95% 4,30–8,41), o Paraná (6,18%; IC95% 4,61–7,76) e o Mato Grosso (6,07%; IC95% 3,77–8,36). Em 2019, as maiores prevalências foram observadas em Mato Grosso (6,23%; IC95% 4,44–8,01), Roraima (6,12%; IC95% 4,46–7,77), Rondônia (5,72%; IC95% 4,10–7,33) e Amapá 5,33% (IC95% 3,37–7,29) (Figura 1A). A análise das RPb mostrou que houve redução significativa dos ATT entre 2013 e 2019 para os estados do Pará (RPb: 0,51; IC95% 0,35–0,76), de Alagoas (RPb: 0,60; IC95% 0,36–0,98) e do Paraná (RPb: 0,65; IC95% 0,44–0,96) e o Distrito Federal (RPb: 0,48; IC95% 0,31–0, 75), além das regiões Norte (RPb: 0,67; IC95% 0,52–0,85) e Nordeste (RPb: 0,81; IC95% 0,68–0,96) (Tabela 1 material suplementar).

**Figura 1:** Prevalência de 2013 e 2019 de adultos brasileiros ocupados que se envolveram em acidente de trabalho nos 12 meses anteriores à entrevista, nos estados (A) e nas capitais (B). Pesquisa Nacional de Saúde 2013 e 2019, Brasil.

Entre as capitais brasileiras, as maiores prevalências de ATT em 2013 foram identificadas em Belém (7,81%; IC95% 3,47–12,14), Porto Velho (7,35%; IC95% 4,46–10,24), Fortaleza (6,49%; IC95% 2,3–10,68) e Boa Vista (6,45%; IC95% 4,29–8,61). Na PNS de 2019, maiores prevalências ocorreram em Fortaleza (7,36%; IC95% 4,2–10,51), Boa Vista (6,57%; IC95% 4,5–8,65), Porto Alegre (6,02%; IC95% 3,66–8,39) e Porto Velho (5,82%; IC95% 3,85–7,79) (Figura 1B). Reduções significativas entre os dois inquéritos foram identificadas em Belém (RPb: 0,45; IC95% 0,21–0,95), Salvador (RPb: 0,42; IC95% 0,23–0,80) e Goiânia (RPb: 0,35; IC95% 0,15–0,79) (Tabela 2 material suplementar).

Em 2019, 4,13% (IC95% 3,80–4,46) da população brasileira ocupada relatou ter sofrido acidente de trabalho nos 12 meses anteriores à pesquisa, sendo que 2,64% (IC95% 2,37–2,91) relataram acidente durante o trabalho e 1,60% (IC95% 1,40–1,80), no deslocamento. Os acidentes ocorridos no local de trabalho foram mais frequentes para o sexo masculino (RPaj: 1,76; IC95% 1,42–2,20), aqueles de 18 a 29 anos (RPaj: 2,22; IC95% 1,53–3,23), 30 a 39 anos (RPaj: 1,70; IC95% 1,19–2,42) e 40 a 59 anos (RPaj: 1,76; IC95% 1,25–2,48), comparados aos com 60 anos ou mais; aqueles sem instrução e com fundamental incompleto (RPaj: 2,64; IC95% 1,89–3,67), com fundamental completo e médio incompleto (RPaj: 2,53; IC95% 1,69–3,77) e com médio completo e superior incompleto (RPaj: 1,87; IC95% 1,35–2,59), comparados aos com superior completo; e as pessoas de cor da pele preta (RPaj: 1,53; IC95% 1,12–2,08) e parda (RPaj: 1,34; IC95% 1,07–1,69), comparadas às pessoas brancas. AT em trabalhadores residentes em zona rural foram mais elevados (RPaj: 1,32; IC95% 1,09–1,60). Os trabalhadores de outras raças (RPaj: 0,44; IC95% 0,24–0,80) e os sem carteira assinada (RPaj: 0,78; IC95% 0,63–0,96) tiveram menor frequência desse tipo de acidente. Já os acidentes ocorridos no deslocamento foram mais frequentes no sexo masculino (RPaj: 2,17; IC95% 1,66–2,84), nas faixas etárias de 18 a 29 anos (RPaj: 4,18; IC95% 2,37–7,37) e de 30 a 39 anos (RPaj: 2,51; IC95% 1,42–4,43), comparadas com a faixa etária de 60 anos ou mais; e nos trabalhadores com ensino fundamental completo e médio incompleto (RPaj: 1,66; IC95% 1,12–2,47), comparados aos com superior completo. Os trabalhadores do Sudeste tiveram menor prevalência (RPaj: 0,59; IC95% 0,41–0,83) em comparação com os trabalhadores do Norte. Os trabalhadores domésticos (RPaj: 0,24; IC95% 0,09–0,62) comparados aos não remunerados e os sem carteira assinada (RPaj: 0,75; IC95% 0,58–0,97) tiveram menor frequência desse acidente (Tabela 2).

**Tabela 2:** Prevalência, razão de prevalência ajustada e IC95% de adultos ocupados que se envolveram em acidentes de trabalho nos 12 meses, segundo variáveis sociodemográficas e de ocupação (n=52.475). Pesquisa Nacional de Saúde 2019, Brasil.

Variáveis			Acidente no trabalho		Acidente no deslocamento		Acidente de trabalho total	
			% (IC95%)	RP_aj_ (IC95%)	% (IC95%)	RP_aj_ (IC95%)	% (IC95%)	RP_aj_ (IC95%)
**Total**			2,64 (2,37–2,91)	-	1,60 (1,40–1,80)	-	4,13 (3,80–4,46)	-
Variáveis sociodemográficas								
	Sexo								
		Masculino	3,27 (2,86–3,67)	**1,76 (1,42**–**2,20)**	2,11 (1,81–2,41)	**2,17 (1,66**–**2,84)**	5,24 (4,76–5,73)	**1,92 (1,62**–**2,27)**
		Feminino	1,86 (1,52–2,19)	1,00 (-)	0,97 (0,74–1,19)	1,00 (-)	2,73 (2,34–3,12)	1,00 (-)
	Faixa etária (anos)							
		18 a 29	3,31 (2,61–4,01)	**2,22 (1,53**–**3,23)**	2,78 (2,20–3,35)	**4,18 (2,37**–**7,37)**	5,78 (4,92–6,65)	**2,71 (1,99**–**3,68)**
		30 a 39	2,50 (2,04–2,97)	**1,70 (1,19**–**2,42)**	1,65 (1,29–1,99)	**2,51 (1,42**–**4,43)**	4,07 (3,50–4,65)	**1,93 (1,43**–**2,60)**
		40 a 59	2,59 (2,18–3,00)	**1,76 (1,25**–**2,48)**	1,11 (0,86–1,36)	1,70 (0,96–3,01)	3,65 (3,18–4,12)	**1,73 (1,29**–**2,32)**
		60 ou mais	1,51 (1,05–1,97)	1,00 (-)	0,68 (0,32–1,03)	1,00 (-)	2,17 (1,59–2,75)	1,00 (-)
	Escolaridade							
		Sem instrução e fundamental incompleto	3,32 (2,83–3,84)	**2,64 (1,89**–**3,67)**	1,44 (1,13–1,74)	1,40 (0,96–2,04)	4,64 (4,07–5,22)	**2,02 (1,58**–**2,58)**
		Fundamental completo/médio incompleto	3,55 (2,55–4,58)	**2,53 (1,69**–**3,77)**	2,24 (1,66–2,81)	**1,66 (1,12**–**2,47)**	5,63 (4,52–6,77)	**2,09 (1,57**–**2,78)**
		Médio completo/superior incompleto	2,56 (2,15–2,98)	**1,87 (1,35**–**2,59)**	1,76 (1,39–2,14)	1,35(0,93–1,97)	4,18 (3,63–4,74)	**1,60 (1,25**–**2,04)**
		Superior completo	1,24 (0,89–1,60)	1,00 (-)	1,05 (0,74–1,36)	1,00 (-)	2,28 (1,82–2,75)	1,00 (-)
	Raça/cor da pele							
		Branca	2,15 (1,79–2,51)	1,00 (-)	1,36 (1,08–1,63)	1,00 (-)	3,38 (2,95–3,82)	1,00 (-)
		Preta	3,35 (2,48–4,25)	**1,53 (1,12**–**2,08)**	1,81 (1,20–2,41)	1,27 (0,87–1,86)	5,00 (3,88–6,11)	**1,43 (1,12**–**1,84)**
		Parda	2,99 (2,56–3,44)	**1,34 (1,07**–**1,69)**	1,80 (1,49–2,11)	1,23 (0,94–1,60)	4,71 (4,20–5,23)	**1,32 (1,12**–**1,57)**
		Outras	0,96 (0,40–1,51)	**0,44 (0,24**–**0,80)**	1,54 (0,03–3,11)	1,08 (0,38–3,08)	2,41 (0,76–4,07)	0,69 (0,34–1,41)
	Local de residência							
		Urbana	2,51 (2,21–2,80)	1,00 (-)	1,64 (1,42–1,86)	1,00 (-)	4,03 (3,67–4,39)	1,00 (-)
		Rural	3,56 (3,03–4,10)	**1,32 (1,09**–**1,60)**	1,35 (0,98–1,71)	0,74 (0,54–1,01)	4,85 (4,22–5,48)	1,11 (0,94–1,30)
	Regiões							
		Norte	2,46 (2,01–2,91)	1,00 (-)	2,43 (1,89–2,97)	1,00 (-)	4,69 (4,00–5,38)	1,00 (-)
		Nordeste	2,57 (2,18–2,95)	1,07 (0,84–1,35)	1,75 (1,42–2,07)	0,75 (0,56–1,00)	4,16 (3,69–4,64)	0,91(0,76–1,10)
		Sudeste	2,60 (2,09–3,10)	1,11 (0,85–1,45)	1,31 (0,95–1,66)	**0,59 (0,41**–**0,83)**	3,80 (3,19–4,42)	0,87 (0,70–1,07)
		Sul	2,85 (2,30–3,38)	1,21 (0,93–1,57)	1,68 (1,27–2,10)	0,75 (0,54–1,05)	4,47 (3,80–5,14)	1,01 (0,82–1,24)
		Centro-Oeste	2,90 (2,22–3,58)	1,21 (0,90–1,63)	1,90 (1,41–2,39)	0,83 (0,59–1,16)	4,66 (3,77–5,55)	1,03 (0,81–1,31)
Variáveis ocupacionais								
	Tipo de trabalho							
		Trabalhador doméstico	1,80 (1,25–2,35)	1,72 (0,75–3,96)	0,39 (0,20–0,58)	**0,24 (0,09**–**0,62)**	2,18 (1,60–2,76)	0,79 (0,41–1,52)
		Militar	1,98 (0,22–4,19)	1,11 (0,28–4,36)	3,41 (0,92–5,89)	0,95 (0,32–2,82)	5,30 (2,02–8,58)	1,02 (0,43–2,42)
		Setor privado	3,28 (2,78–3,78)	2,15 (0,97–4,79)	2,06 (1,71–2,42)	0,70 (0,31–1,58)	5,18 (4,60–5,78)	1,19 (0,64–2,19)
		Setor público	1,96 (1,30–2,62)	1,52 (0,65–3,58)	0,97 (0,69–1,25)	0,47 (0,20–1,10)	2,93 (2,22–3,65)	0,85 (0,44–1,62)
		Empregador	1,75 (0,95–2,55)	1,21 (0,49–3,02)	1,01 (0,01–2,01)	0,43 (0,12–1,49)	2,71 (1,46–3,97)	0,69 (0,32–1,48)
		Conta própria	2,31 (1,97–2,66)	1,61 (0,72–3,58)	1,43 (1,11–1,74)	0,58 (0,25–1,34)	3,61 (3,15–4,08)	0,91 (0,49–1,69)
		Não remunerado	1,27 (0,28–2,26)	1,00 (-)	2,42 (0,46–4,38)	1,00 (-)	3,59 (1,42–5,76)	1,00 (-)
	Carteira de trabalho assinada							
		Sim	3,19 (2,64–3,74)	1,00 (-)	2,06 (1,65–2,47)	1,00 (-)	5,09 (4,44–5,75)	1,00 (-)
		Não*	2,31 (2,04–2,58)	**0,78 (0,63**–**0,96)**	1,33 (1,12–1,53)	**0,75 (0,58**–**0,97)**	3,56 (3,22–3,89)	**0,77 (0,66**–**0,90)**

Obs.: Os dados faltantes não foram apresentados.

* Dados faltantes foram classificados como categoria “Não”; valores em negrito: estatisticamente significativos (p<0,05). RP_aj:_ razão de prevalência ajustada por sexo e idade; IC95%: intervalo de confiança de 95%.

No que se refere aos ATT, estiveram positivamente associados a: sexo masculino (RPaj: 1,92; IC95% 1,62–2,27), faixas etárias mais jovens ([18 a 29 anos – RPaj: 2,71; IC95% 1,99–3,68], [30 a 39 anos – RPaj: 1,93; IC95% 1,43–2,60], [40 a 59 anos – RPaj: 1,73; IC95% 1,29–2,32]), em relação à de 60 anos ou mais; menores escolaridades ([sem instrução e fundamental incompleto – RPaj: 2,02; IC95% 1,58–2,58], [fundamental completo e médio incompleto – RPaj: 2,09; IC95% 1,57–2,78], [médio completo e superior incompleto – RPaj: 1,60; IC95% 1,25–2,04]), em relação ao ensino superior completo; e cores da pele preta (RPaj: 1,43; IC95% 1,12–1,84) e parda (RPaj: 1,32; IC95% 1,12–1,57), comparadas com a raça branca. Na contramão, ATT se associou de forma negativa à ausência de carteira de trabalho (RPaj: 0,77; IC95% 0,66–0,90) em relação àqueles que a tinham (Tabela 2).

## DISCUSSÃO

Este estudo apontou uma redução dos ATT entre 2013 e 2019. Em 2019, as principais vítimas de ATT foram homens, jovens, pretos e pardos, indivíduos menos escolarizados e com carteira assinada. Os acidentes de trabalho típicos foram mais frequentes entre os trabalhadores rurais e os acidentes de deslocamento não tiveram diferença entre urbano e rural. Em 2019, o relato de acidentes de trabalho ocorreu em cerca de 4% da população ativa, sendo que os acidentes de deslocamento para o trabalho correspondem a cerca de 38% dos acidentes totais. Em 2013, o Pará liderou os ATT e, em 2019, a maior prevalência foi em Mato Grosso.

Foram apontadas disparidades entre UF, com piores resultados no Pará, em Roraima, em Mato Grosso e em Mato Grosso do Sul. Dados do Ministério da Previdência e Assistência Social destacam piores resultados de acidentes de trabalho em estados do Norte e Nordeste do país^13^. Entretanto, na comparação entre 2013 e 2019, essas duas regiões apresentaram reduções significativas nas prevalências, enquanto os estados do Centro-Oeste se mantiveram na liderança. Essa alta prevalência de ATT no Centro-Oeste pode ser explicada pelo grande número de trabalhadores rurais e pela elevada ocorrência de acidentes de trabalho na zona rural. Os acidentes ocupacionais envolvendo máquinas e equipamentos resultam em amputações e outras lesões graves com frequência 15 vezes maior do que as demais causas, gerando três vezes mais acidentes fatais que a média geral^4^.

Mato Grosso é o segundo estado com maior prevalência de notificação de acidentes, com 150 casos a cada 10 mil trabalhadores, atrás apenas do Rio Grande do Sul. O estado registrou mais de 10 mil acidentes de trabalho em 2021 e, nos últimos 10 anos (2012 a 2021), grande parte dos acidentes foi causada pela operação de máquinas e equipamentos (15%)^4^. Em relação a mortalidade, Mato Grosso ocupa a primeira posição com 14 óbitos por 10 mil trabalhadores^4^. Esse perfil de acidentalidade pode estar relacionado ao perfil produtivo do estado, no qual a agropecuária emprega em torno de 70% da população economicamente ativa dos municípios^14^.

Esse cenário mostra a necessidade de capacitação adequada dos trabalhadores e maior fiscalização quanto ao cumprimento das normas de segurança, em especial, da Norma Regulamentadora n° 12 (NR-12) do Ministério do Trabalho e Previdência^15^, sobre Segurança no Trabalho em Máquinas.

A PNS de 2019 identificou que cerca 38% dos ATT ocorreram no deslocamento, percentual maior que o encontrado na PNS de 2013 (30%)^10^. De acordo com o previsto no art. 21, inciso IV, alínea *d*, da Lei n° 8.213/1991, o acidente de deslocamento assegura ao trabalhador acidentado os mesmos direitos de acidente de trabalho típico^1^. Entretanto, em 12 de novembro de 2019, a Medida Provisória (MP) n° 905^16^ estabeleceu que o acidente de deslocamento não deveria conferir ao empregado os mesmos direitos que o acidente de trabalho. Por ser provisória, essa MP não foi convertida em lei e expirou a partir de 21 de abril de 2020. Com isso, a flexibilização pretendida também deixou de existir e, consequentemente, o acidente de deslocamento e acidente de trabalho são iguais perante a lei^1^.

Dados de atendimentos de emergência por lesões relacionadas ao trabalho também apontam que, entre os acidentes de trabalho, 31,3% estavam relacionados ao transporte^17^. É importante destacar que o aumento das atividades envolvendo motocicleta, usadas para transporte/deslocamento entre o trabalho e a residência e como instrumento de trabalho (mototáxi, entregas de mercadorias, entre outros), pode ter contribuído para o incremento desses acidentes. Soma-se a isso o fato de que houve um crescimento de 53% das mortes de motociclistas de 1990 a 2019, sendo que a taxa de mortalidade para os homens aumentou de 7,3/100.000 (1990) para 11,7/100.000 habitantes (2019)^18^.

Os achados da PNS 2019 estão em concordância com outros estudos que apontam que homens, jovens (18 a 39 anos), pardos e pretos, trabalhadores com menor escolaridade e menor renda são mais expostos aos acidentes^3,17,19–21^.

A literatura aponta que os setores com maiores índices de AT são da indústria e da construção civil, que empregam mais trabalhadores do sexo masculino, com baixa qualificação e com menor escolaridade, portanto com maior vulnerabilidade social e mais acidentalidade^4,22–24^. A maior acidentalidade entre jovens pode estar relacionada a menor experiencia no oficio, menor qualificação, além de exposição a empregos de maior rico, como a construção civil^25^.

O estudo apontou que a prevalência de AT na zona rural foi cerca de 30% mais elevada que na urbana, entretanto os AD não tiveram diferenças entre as zonas urbana e rural. Estudos anteriores apontaram maior prevalência de acidentes de trabalho na zona rural^25,26^, o que pode ser explicado pelas especificidades desse trabalho, com riscos inerentes ao manejo de animais, maquinários, exposição a materiais cortantes, contaminados e, muitas vezes, esses riscos são acrescidos pela baixa escolaridade e capacitação dos trabalhadores. Destaca-se a importância da PNS para esse registro, pois as notificações de acidentes na zona urbana historicamente são mais elevadas, o que se deve ao maior acesso aos serviços de saúde na zona urbana, combinado com o trabalho informal no campo, levando à subnotificação e à minimização dos riscos de acidentes^26^.

A maior ocorrência de ATT em trabalhadores com carteira assinada está de acordo com estudos anteriores^27,28^. No entanto outros estudos relatam maior prevalência de ATT entre trabalhadores com contrato informal de trabalho (54,3%)^19^. Portanto esses resultados precisam ser melhor investigados, considerando outras variáveis do processo saúde-doença-trabalho.

Estudo comparando dados oficiais do Ministério da Previdência Social e os divulgados pela PNS em 2013 verificou que a PNS encontrou sete vezes mais acidentes de trabalho do que os dados sobre acidentes registrados pela Previdência^13^. Dados do ministério incluem apenas os trabalhadores com vínculo formal, excluindo os trabalhadores informais^13,17^, portanto monitorar ATT pela PNS torna-se fundamental.

No entanto, desde 2015, o Brasil tem experimentado um cenário de crises para os trabalhadores, com aumento do desemprego, redução da proteção garantida pelas políticas públicas e adoção de medidas de austeridade^7^. A Reforma Trabalhista, sancionada em 13 de julho de 2017 pelo Presidente Michel Temer, Lei n° 13.467^29^, flexibilizou as relações de trabalho, aumentou o número de trabalhadores na informalidade e em trabalho precário^8^. A grande parcela de trabalhadores informais não é contabilizada nas estatísticas oficiais, o que pode tornar os ATT ainda mais subnotificados^30^.

Por último, ressalta-se que os dados da PNS 2019 foram colhidos no ano anterior à pandemia da COVID-19. A crise sanitária pode ter afetado a ocorrência de ATT, uma vez que o aumento da informalidade, além de trabalhadores de entrega por bicicleta e motos, pode ter impactado diretamente a segurança do trabalho^31,32^.

Entre os limites do estudo, apontam-se aqueles inerentes aos estudos transversais, como, por exemplo, a limitação na determinação de causalidade. Além disso, os dados são autorreferidos, sendo possível ocorrerem diferenças na compreensão dos entrevistados, viés recordatório e sub ou superestimação dos valores referidos que podem ter influenciado os resultados.

Este é o primeiro estudo com a comparação dos dados de ATT entre as PNS 2013 e 2019, possibilitando o reconhecimento da situação desse agravo no país e sua evolução nos últimos anos. O tamanho amostral de abrangência representativa da população adulta brasileira e os procedimentos de pesquisa adotados fortalecem a confiabilidade dos dados.

Em conclusão, esse estudo mostrou que houve uma redução dos ATT no Brasil entre 2013 e 2019, com importantes vulnerabilidades para esse agravo entre homens, pardos e pretos, jovens e com menor escolaridade. Os acidentes de trabalho típicos foram até 32% mais elevados na zona rural e cresceram os acidentes por deslocamento em 2019. Visto que os direitos trabalhistas e a segurança no trabalho fazem parte dos Objetivos do Desenvolvimento Sustentável da Agenda 2030^33^, o presente estudo possibilita a identificação de grupos prioritários para a construção de agendas de ações direcionadas à prevenção dos acidentes de trabalho. Dessa forma, é necessário que haja uma colaboração intersetorial que vise não somente a redução de custos para a economia e previdência social, mas principalmente o bem-estar dos trabalhadores e a equidade social.
